# Comparative evaluation of double- and single-armed two-suture longitudinal intussusception techniques in microsurgical vasoepididymostomy: An updated systematic review and meta-analysis

**DOI:** 10.1371/journal.pone.0298019

**Published:** 2024-02-05

**Authors:** Hong Xiao, Shan Zhou, Qiang Chen, Yilang Ding, Peng Yang, Hailin Huang, Xi Chen, Huiliang Zhou, Songxi Tang

**Affiliations:** Department of Andrology and Sexual Medicine, First Affiliated Hospital of Fujian Medical University, Fuzhou, China; Shanghai Jiao Tong University Medical School Affiliated Ruijin Hospital, CHINA

## Abstract

**Background:**

This study aimed to compare the outcomes of double−armed two−suture longitudinal intussusception microsurgical vasoepididymostomy (LIVE) and single−armed two−suture LIVE techniques in patients with epididymal obstructive azoospermia (EOA). The main outcomes assessed were patency rates, patency time, semen quality and natural pregnancy rates.

**Methods:**

Data from patients with EOA who underwent two−suture LIVE were obtained from databases including PubMed, EMBASE, and Web of Science. Weighted data were analyzed using a random−effects model, and weighted mean differences were reported.

**Results:**

A total of 1574 patients with EOA from 24 studies were included. The overall patency rate was approximately 68% (95% confidence interval [CI]: 63–72%), with a patency time of approximately 4.63 months (95% CI: 4.15–5.12). The sperm concentration reached 26.90 million/ml and the sperm motility was 23.74%. The natural pregnancy rate was 38% (95% CI: 31–46%). The different definitions of patency do not seem to have any meaningful impact when comparing patency rates. There was no significant difference in patency rates, patency time, semen quality and natural pregnancy rates between the double−armed and single−armed LIVE techniques.

**Conclusion:**

The single−armed LIVE is a potential alternative surgical option when high quality double−needle sutures are not easily accessible.

## Introduction

Microsurgical vasoepididymostomy (MVE) is the preferred surgical approach for treating epididymal obstructive azoospermia (EOA). Compared to artificial reproductive technology (ART), MVE has distinct advantages, such as the absence of ethical concerns and being a more cost-effective means of achieving successful reproduction. Over the past 40 years, many surgical techniques for MVE have been developed, including end-to-end anastomosis, end-to-side anastomosis, three-suture triangulation intussusception, two-suture transverse intussusception vasoepididymostomy, and two-suture double-armed longitudinal intussusception vasoepididymostomy (DA-LIVE).

Currently, the traditional two-suture DA-LIVE technique has become the standard approach for vasoepididymostomy due to its widespread adoption [1]. However, the limited availability and high cost of double-armed sutures outside the United States pose a significant challenge to the application of DA-LIVE. To address this challenge, an alternative technique called single-armed longitudinal intussusception microsurgical vasoepididymostomy (SA-LIVE) was introduced by Monoski in 2007 [2]. This technique utilizes single-needle sutures, which are more accessible and cost-effective, and achieves comparable patency rates to the DA-LIVE technique.

In recent years, the surgical technique of SA-LIVE has gained increasing popularity in certain countries, including China [3–11]. A prior meta-analysis comparing patency rates between DA-LIVE and SA-LIVE found slightly higher patency rates in the SA-LIVE group [12]. However, it is important to approach these conclusions cautiously and interpret them carefully. The previous analysis did not delve into the reasons for the observed differences between the two techniques or compare other critical factors, such as postoperative patency time and semen parameters. Consequently, despite the effectiveness of DA-LIVE and SA-LIVE in treating EOA, significant controversies persist regarding various surgical procedures and their outcomes.

In our study, we conducted a comprehensive analysis to compare the efficacy of two distinct microsurgical techniques for addressing EOA: the two-suture DA-LIVE and SA-LIVE. Our analysis extended beyond the basic examination of postoperative indicators, such as patency and pregnancy rates in partners. We also investigated patency time and post-patency semen parameters to provide a more thorough evaluation of the outcomes associated with LIVE in managing EOA. Through systematic reviews and meta-analyses, our objective was to assess the effectiveness of different treatment strategies, providing valuable reference information and enhancing our understanding of the outcomes and implications of LIVE.

## Materials and methods

### Literature search

We adhered to the Preferred Reporting Items for Systematic Reviews and Meta-Analyse (PRISMA) guidelines when conducting our study. The study protocol was published in PROSPERO (registration number: CRD42023450020).

Two independent authors (Hong Xiao and Songxi Tang) searched PubMed, EMBASE, and Web of Science databases to obtain all relevant data up until June 2023 using the following search terms: (“azoospermia”) OR (“epididymal obstructive azoospermia”) OR (“epididymal obstruction”) OR (“epididymis obstruction”) OR (“vasoepididymal obstruction”) OR (“infertility”) OR (“fertility”)) AND (“vasoepididymostomy”) OR (“epididymovasostomy”).

### Study selection and data extraction

The selected studies met the following inclusion criteria: (i) included participants diagnosed with EOA who underwent two-suture LIVE; (ii) controlled trials, cohort studies, case-control studies, cross-sectional surveys, and descriptive studies; (iii) original human studies; (iv) included patency as an outcome measure; and (v) were published before June 2023.

The exclusion criteria were: (a) reviews, conference abstracts, animal experiments, and case reports with <10 cases; (b) absence of two-suture intussusception vasoepididymostomy for treating EOA; and (c) inclusion of overlapping participants.

Two authors independently evaluated the search results. Huiliang Zhou, the senior author, thoroughly assessed the articles to confirm their relevance to the study and ultimately determined which ones would be included. The search strategy was initially created for PubMed and then adjusted for the other databases. The following data were obtained from relevant articles: publication years, authors’ names, methods used in the study design, surgical techniques employed, sample sizes, ages of the patients, patency rates, patency duration, pregnancy rates, follow-up duration, and semen quality.

### Quality assessment

The Quality Assessment Tool for Before-After (Pre-Post) Studies with No Control Group developed by the National Institutes of Health (NIH) was used to evaluate the quality of the included uncontrolled before-after studies. The tool consists of 12 items that assess the risk of bias in six domains: selection bias, study design, confounders identification and adjustment, blinding, data collection methods, and withdrawals and dropouts. Each item can be rated as "yes," "no," "not reported," or "not applicable" based on the information provided in the study. The overall quality of each study is then classified as "good," "fair," or "poor," depending on the number of "yes" responses.

In our analysis, we applied the tool to evaluate the quality of studies that met our inclusion criteria. Specifically, we checked if the study had a clear description of the intervention and outcome measures, if the study population was well-defined and representative of the target population, if the data collection method was reliable and valid, if the study accounted for potential confounding factors, if the outcome assessment was valid and reliable, and if the study reported sufficient data to allow for a meta-analysis. However, in this analysis, the 12th item must always be marked as "Not applicable," leading to a maximum potential quality score of 11.

Two reviewers (Hong Xiao and Songxi Tang) independently assessed the quality of the included studies using the tool. Any discrepancies were resolved by discussion and consensus. The results of the quality assessment are presented in **Table 1**.

**Table 1:** Characteristics of the 24 records included in the meta-analysis.

Study	Design	Surgical technique	N	Age (range)	Patency time in months (range)	Patency rate, %	Pregnancy rate, %	Follow-up in months (range)	Sperm concentration, million/ml (range)	Sperm motility % (range)	Quality score*
Schiff, J. et al.	retrospective	DA-LIVE	15	30.4	2.9 (1–6)	80	44	17.2	17.7	19	9
Kumar, R. et al.	Prospective	DA-LIVE	23	30.7 (24–38)	3.2 (1.5–7)	48	N/A	7.6 (1.5–30)	17(10–65)	N/A	5
Ho, K. L. et al.	Retrospective	DA-LIVE	17	N/A	N/A	53	N/A	N/A	N/A	N/A	8
Zhang, G. X. et al.	Retrospective	DA-LIVE	42	37	N/A	71.4	33.3	>6–12	N/A	N/A	7
Kumar, R. et al.	Prospective	DA-LIVE	23	31 (23–40)	6.6 (3–15)	48	9.09	11.47 (3–26)	18 (0.1–90)	0–50	7
Peng, J. et al.	Retrospective	DA-LIVE	72	30.4 (21–57)	N/A	63.9	47.8	24 (11–45)	20.3(3–200)	0–54	6
Peng, J. et al.	Retrospective	DA-LIVE	53	31(23~48)	4 (3–9)	71.7	44.7	15 (4–22)	1–48	0–65	9
Zhao, L. et al.	Retrospective	SA-LIVE	17	30.4	N/A	58.8	N/A	N/A	12.2	N/A	6
Binsaleh, S. et al.	Retrospective	SA-LIVE	22	31 (23–47)	3 (1–24)	59	23.1	18 (6–30)	29.7 (0–89)	23 (0–57)	7
Peng, J. et al.	Retrospective	DA-LIVE	53	30.4 (22–48)	3.6 (3–7)	79.2	45.2	19.8 (6–43)	38.6 (5–120)	6–64	9
Zhang, F. B. et al.	Retrospective	SA-LIVE	76	N/A	N/A	59.2	17.7	5 (2–16)	28.2	20.5	7
Zhao, L. et al.	Retrospective	SA-LIVE	39	31.4	6.2 (1.5–12)	61.5	62.5	10.3 (2.5–12)	42.1 (0.7–103)	10.9 (0–28)	7
Chen, X. F. et al.	Prospective	SA-LIVE	150	28.5 (22–38)	N/A	72	53.7	16.5 (4–28)	N/A	N/A	8
Hong, K. et al.	Retrospective	SA-LIVE	62	31 (23–45)	N/A	66.1	34.1	8.8 (2–17)	17.1 (0–51.8)	24 (0–52)	8
Peng, J. et al.	Retrospective	DA-LIVE	198	31.0 (20–51)	3.8 (2–10)	76.3	53.6	25.3 (12–48)	N/A	N/A	9
Lyu, K. L. et al.	Retrospective	SA-LIVE	59	31.1 (18–42)	N/A	83.1	49.0	15.6 (3–33)	25.8 (0.8–100)	25 (0–80)	8
Tang, S. X. et al.	Retrospective	SA-LIVE	69	25 (21–42)	4.5 (2–18)	72.5	34.0	12.0 (3–29)	29 (0.1–137.2)	29.9 (0–57.9)	7
Li, J. P. et al.	Retrospective	SA-LIVE	84	31.26	4.3	60.71	54.9	N/A	N/A	N/A	9
Shimpi, R. K. et al.	Prospective	DA-LIVE	40	30.2 (24–37)	N/A	50	30.0	12	N/A	N/A	7
Hibi, H. et al.	Retrospective	DA-LIVE	45	33.2 (25–44)	6.5(0.7–15)	55.6	28.0	N/A	12.5 (0.1–45)	33.2 (0–70)	8
Liu, N. et al.	Retrospective	SA-LIVE	134	32.1 (23–50)	4.1	55.2	27.0	17 (3–36)	N/A	N/A	8
Li, P. et al.	Retrospective	SA-LIVE	40	30.4	3.6	82.5	51.5	16.9 (12–23)	16.8	18.7	9
Zhou, M. K. et al.	Retrospective	SA-LIVE	82	30.77 (20–47)	4.63 (1–12)	81.7	35.8	N/A	N/A	N/A	8
Wang, S. Y. et al.	Retrospective	SA-LIVE	159	N/A	N/A	73	N/A	27.7 (12–48)	N/A	N/A	7

DA-LIVE: double-armed intussusception vasoepididymostomy; SA-LIVE: single-armed intussusception microsurgical vasoepididymostomy; N/A: not applicable

### Statistical analysis

An event rate estimation was performed using a random-effects model. To assess heterogeneity, the Q statistic and I^2^ values were examined. A P-value of the Q statistic <0.10 implied no homogeneity. The pooled results were analyzed using the random-effects model, and sensitivity analysis was conducted by excluding individual studies to evaluate the stability of the results. A fixed-effects model was employed when no heterogeneity was observed. Potential publication bias was evaluated using funnel plots and Egger’s test. Review Manager 5.3 (Cochrane Collaboration, Oxford, UK) software was used to conduct the meta-analysis and perform Egger’s test. For data mentioned in the records that approximately follows a normal distribution, the method suggests approximating the median as the mean and estimating the standard deviation as the quartile range divided by (2 × 1.349).

## Results

### Eligible studies

**Fig 1:**
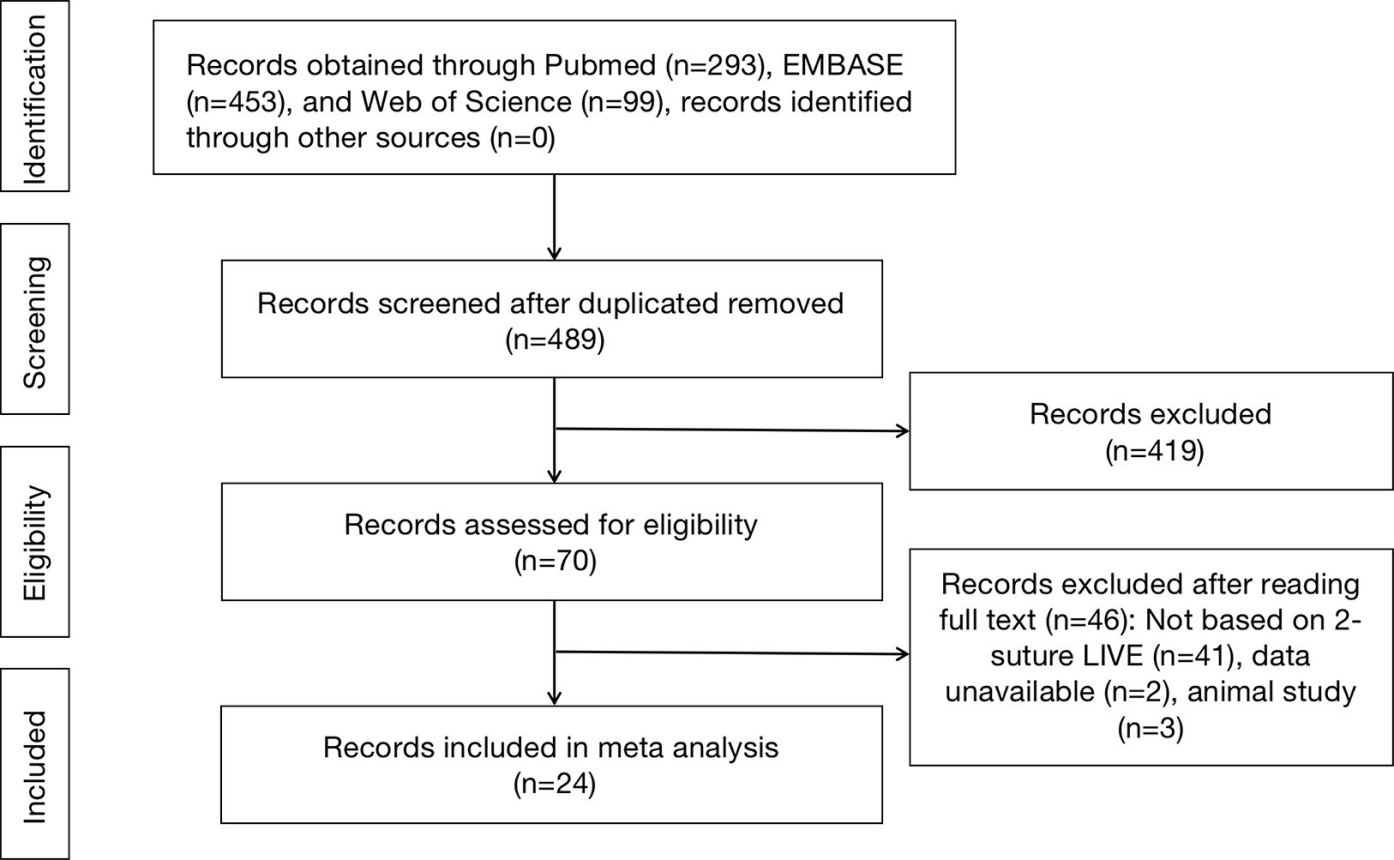
Flow diagram of the included studies in this meta-analysis.

Of the 489 articles identified, 24 studies were eligible and included in the meta-analysis [3–10, 13–29]. This study’s identification and selection process is illustrated in **Fig 1 (S1 and S2 Files)**, according to the PRISMA flow chart. These 24 studies included 1574 patients. The data from these studies were qualitatively and quantitatively analyzed (**Table 1**).

### Quality assessment and publication bias

**Fig 2:**
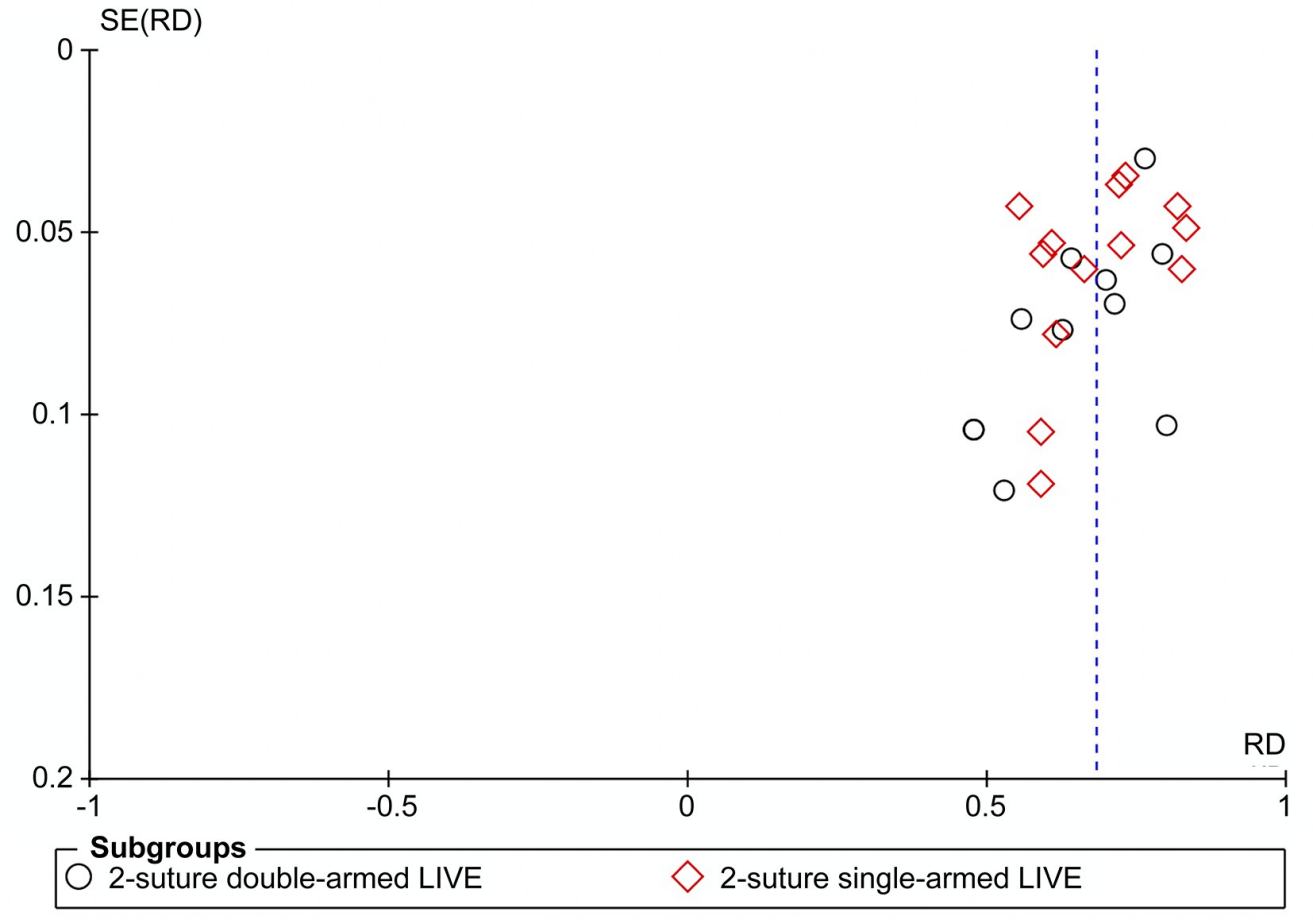
Publishing bias: Funnel plots for patency.

Table 1 presents the evaluation results using the "Quality Assessment Tool for Before-After (Pre-Post) Studies with No Control Group." The median quality score was 8 (ranging from 5–9). We assessed publication bias using Egger’s test and by examining funnel plots (**Fig 2**). Based on Egger’s test results, publication bias was observed in the overall patency rate (*P*<0.05). To address this bias, we assumed that the missing or unpublished studies have similar characteristics and effect sizes to those that were included in the meta−analysis. We also assumed that publication bias is primarily due to the preference for significant or positive results. In order to account for publication bias, we employed the cut−and−patch method to add dummy literature and retested the results. However, the *P*−value remained >0.05, suggesting that our findings were robust and reliable. The results indicate that the added dummy literature did not substantially affect the overall results, thus supporting the robustness of our findings and indicating a limited influence of publication bias.

### Patency rate

**Fig 3:**
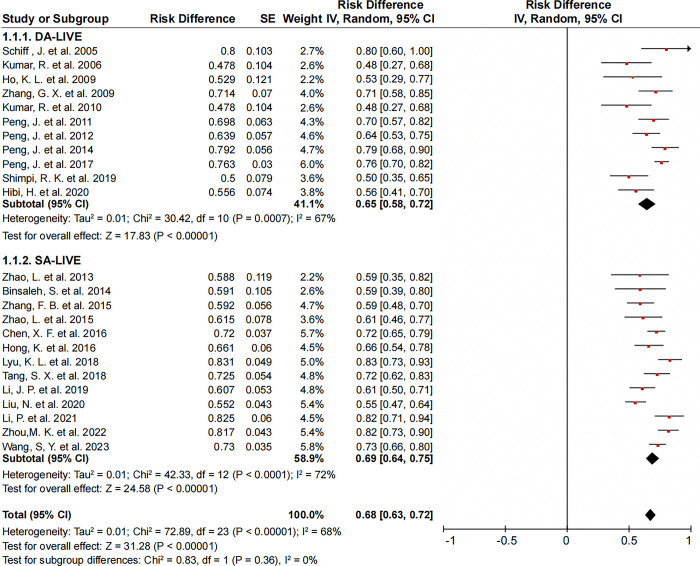
Forest plots of patency rate under DA-LIVE and SA-LIVE. (a random-effects model was used).

Overall, 24 records comprising 581 and 993 patients who underwent DA-LIVE and SA-LIVE, respectively, were included in the analysis of the patency rate. The overall mean patency rate was 68% (95% confidence interval [CI], 63–72%), while the mean patency rate for DA-LIVE and SA-LIVE was 65% (95% CI, 58–72%) and 69% (95% CI, 64–75%), respectively (**Fig 3**). Heterogeneity was relatively high (I^2^ = 68%, *P*<0.01). The relatively high heterogeneity observed in the study results could potentially be caused by several factors, including variations in patency criteria, surgical techniques, sample size, follow-up duration, and even patient characteristics. To gain a deeper understanding of the reasons behind this heterogeneity, we conducted additional analyses specifically focusing on the influence of patency criteria and surgical technologies.

**Fig 4:**
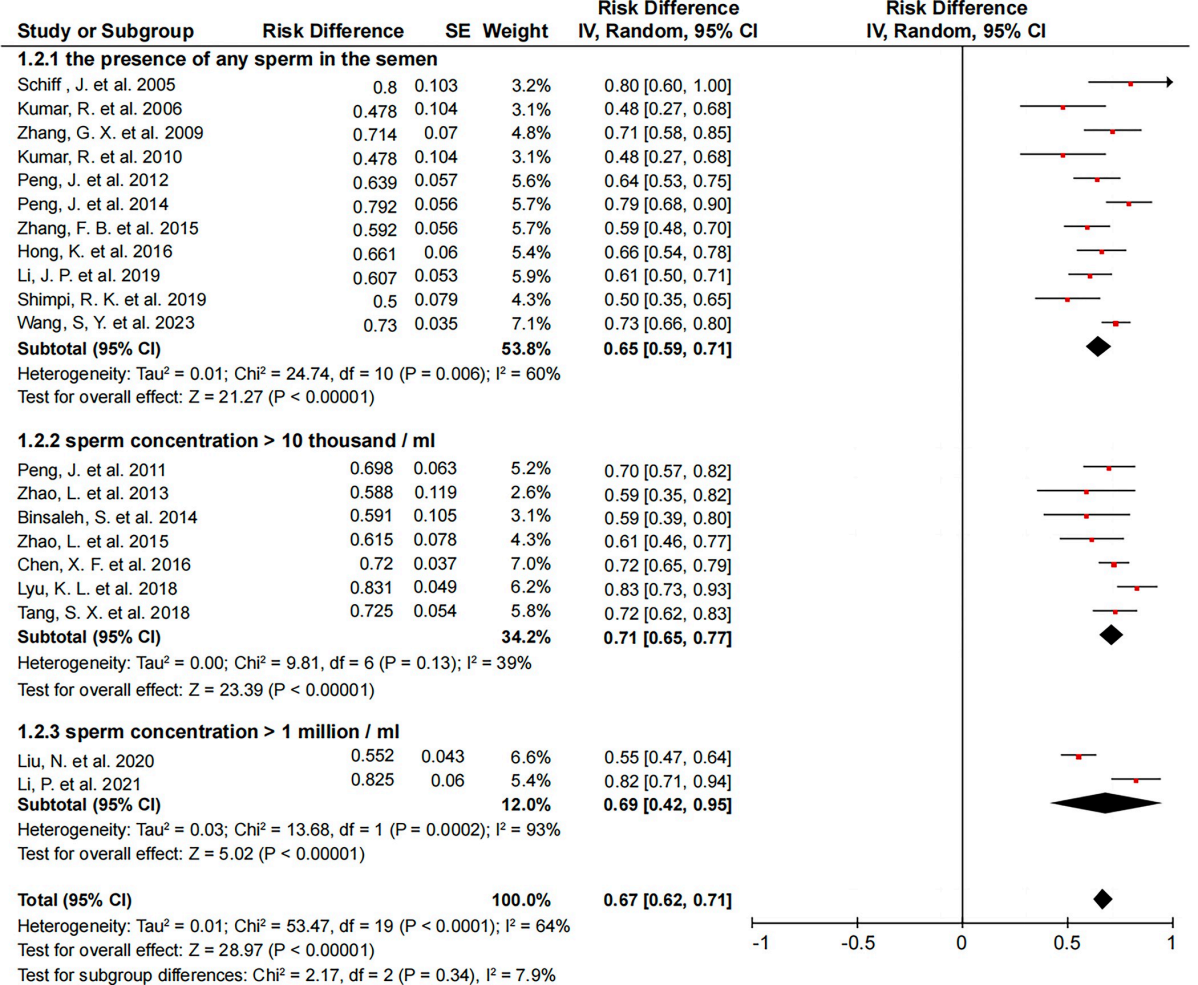
Forest plots of different patency criteria to compare the patency rate (random-effects model was used).

**Fig 4** shows different patency criteria for the patency rate. The mean patency rate was 65% (95% CI, 59–71%) when the patency criterion was the presence of any sperm in the semen [3, 6, 13, 16, 17, 20, 22, 24, 27, 28, 30]. When the patency criterion was sperm concentration >10 thousand/ml [4, 5, 11, 15, 21, 25, 26] or >1 million /ml [7, 8], the mean patency rate was 71% (95% CI, 65–77%) and 69% (95% CI, 42–95%), respectively. No significant difference was observed between patency criteria and patency rate.

**Fig 5:**
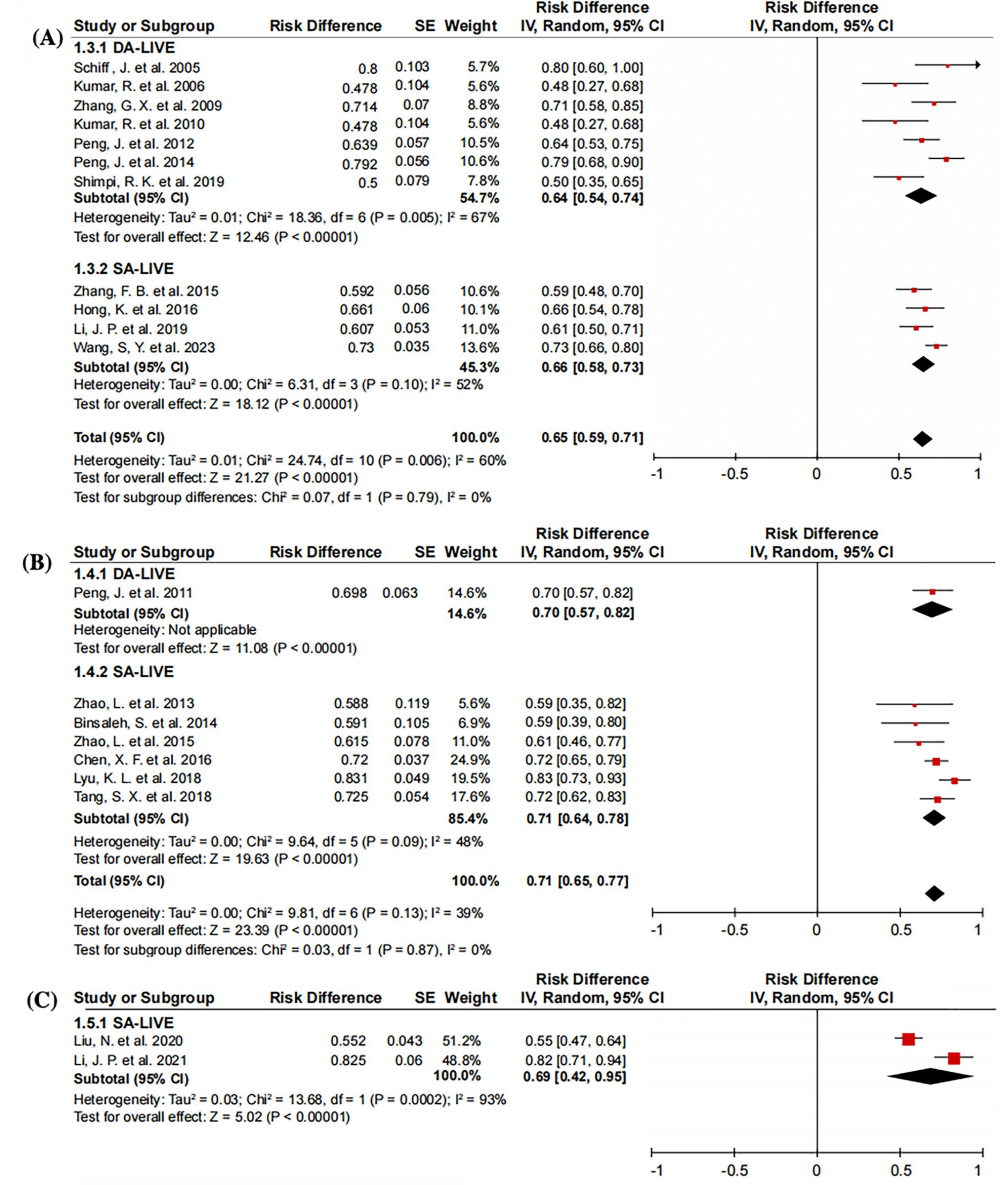
Forest plots of different patency criteria to compare the patency rate of DA-LIVE and SA-LIVE. (A) represents the forest plots where patency was defined as the presence of any sperm in the semen. (B) and (C) represent the forest plots where patency was defined as sperm concentration >10 thousand/ml and >1 million/ml (a random-effects model was used).

By classifying different patency criteria, the patency rate was not significantly different between DA-LIVE and SA-LIVE (**Fig 5**). However, some studies have set the standard for patency at 1 million/ml [7, 8]. Nevertheless, as the surgical technique used was the SA-LIVE alone, comparisons between DA-LIVE and SA-LIVE could not be made.

### Patency time

**Fig 6:**
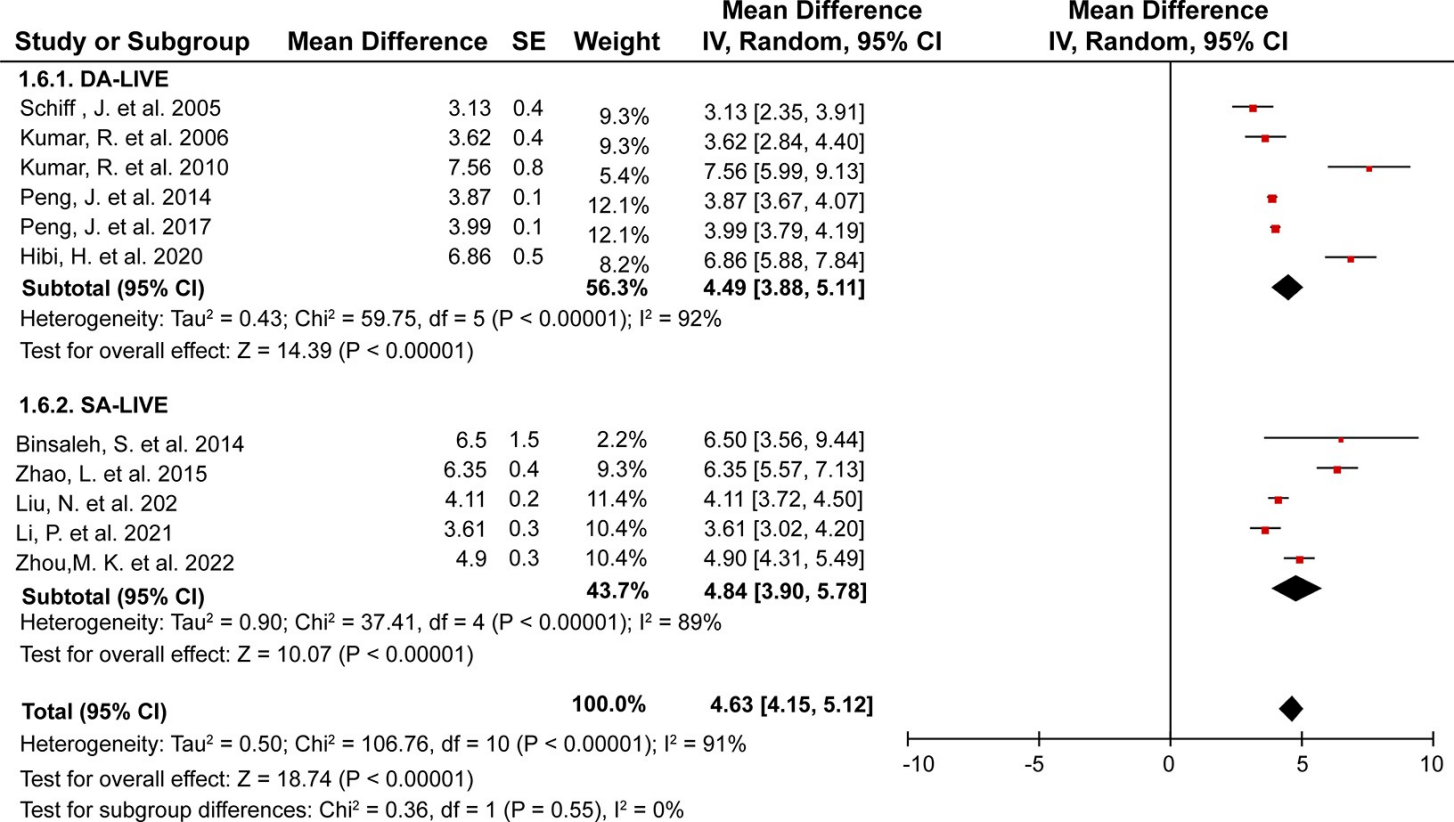
Forest plots of patency times under DA-LIVE and SA-LIVE (random-effects model for eleven studies).

Eleven records containing 357 patients with EOA who underwent DA-LIVE and 317 patients who underwent SA-LIVE were involved in the analysis of patency time between the two surgery types. The overall mean patency time was 4.63 months (95% CI, 4.15–5.12; **Fig 6**). The results of the study revealed that there was no statistically significant difference in patency time between DA-LIVE and SA-LIVE techniques. The mean difference, with a 95% CI, was 4.49 (3.88, 5.11) for DA-LIVE and 4.84 (3.90, 5.78) for SA-LIVE, with a *P*-value >0.05.

### Semen parameters

**Fig 7:**
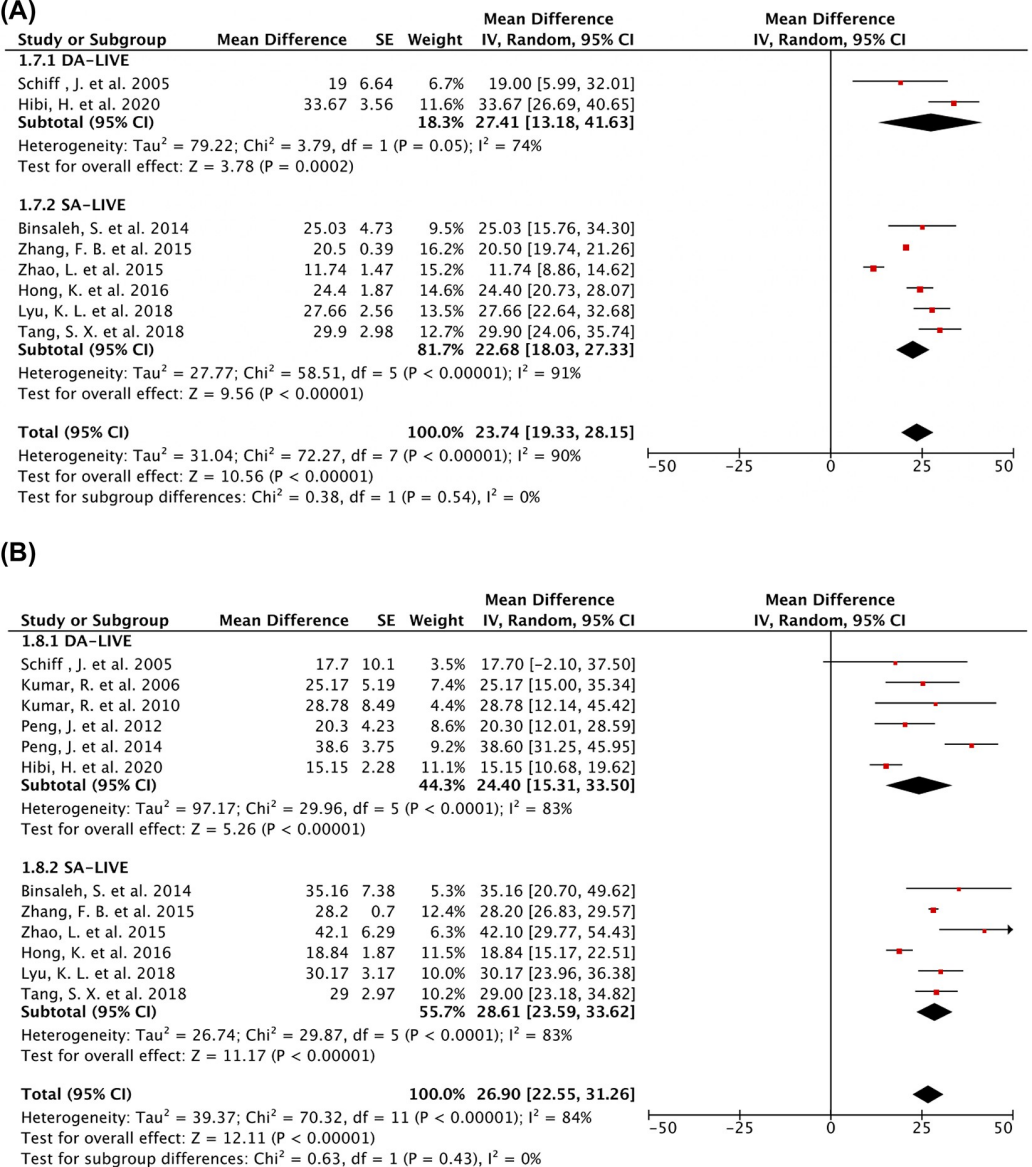
Forest plots of semen parameters under DA-LIVE and SA-LIVE. (A) and (B) represent the forest plots of sperm motility and sperm concentration (random-effects model for eight and twelve studies, respectively).

Eight studies reported sperm motility. DA-LIVE and SA-LIVE were performed in 60 and 327 patients with EOA, respectively. Twelve studies in which sperm concentration was reported included 231 patients who underwent DA-LIVE and 327 who underwent SA-LIVE. The mean sperm motility and concentration were 23.74% (95% CI, 19.33–28.15) and 26.90 million/ml (95% CI, 22.55–31.26), respectively (**Fig 7**). No significant sperm motility difference was observed between patients that underwent DA-LIVE and SA-LIVE (mean difference [95% CI], 27.41 [13.18, 41.63] vs. 22.68 [18.03, 27.33]; *P*>0.05) and sperm concentration (mean difference [95% CI], 24.40 [15.31, 33.50] vs. 28.61 [23.59, 35.62]; *P*>0.05).

### Natural pregnancy rate

**Fig 8:**
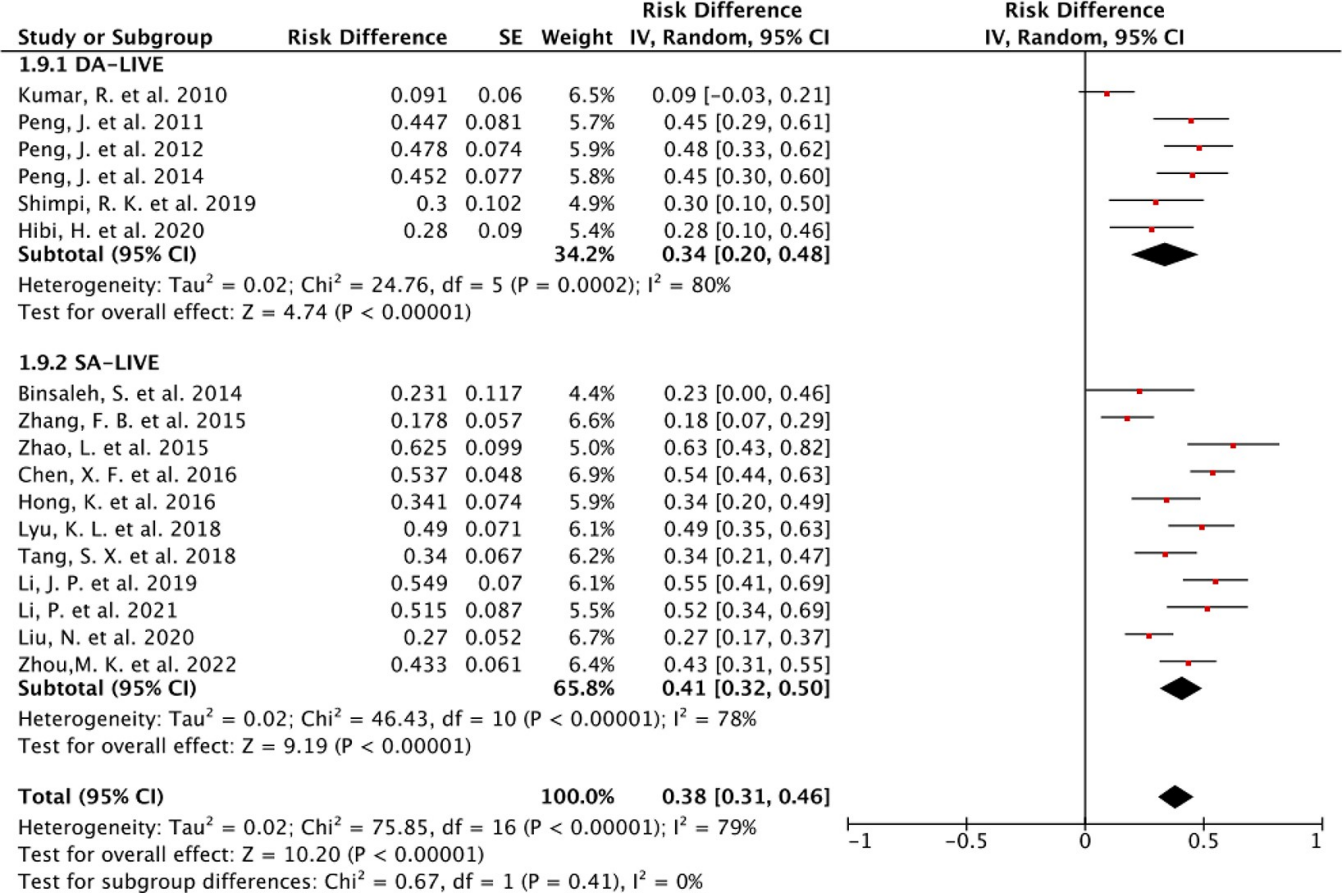
Forest plots of pregnancy rates under DA-LIVE and SA-LIVE (random-effects model for seventeen studies).

In this study, the natural pregnancy rate was strictly defined as the ratio of the number of pregnancies in patients’ partners to the number of successfully restored patency cases, excluding ART-induced pregnancies. Four articles [10, 13–15] had no data on pregnancy rates. Additionally, three other articles [16–18] did not clearly define the calculation of pregnancy rates. Hence, the meta-analysis included 1103 participants from 17 articles, focusing solely on the natural pregnancy rate. Our study exhibited relatively high heterogeneity (I^2^ = 79%, *P*<0.01). The overall mean natural pregnancy rate was 38% (95% CI, 31–46%) (**Fig 8**). The mean natural pregnancy rates obtained from studies involving DA-LIVE and SA-LIVE were 34% (95% CI 20–48%) and 41% (95% CI 32–50%), respectively. No statistical difference was observed in natural pregnancy rates between the two techniques (**Fig 8**).

## Discussion

The LIVE technique, developed by Chan and his colleagues from Cornell University, has revolutionized the field of microsurgical treatment for male infertility. [29] Our comprehensive analysis of 24 studies involving 1574 patients with EOA revealed a considerable overall patency rate of approximately 68% (95% CI, 63‒72%) for LIVE. Similar findings were obtained from other studies that reported high patency rates for LIVE [12, 30]. In comparison, the MVE techniques, such as end-to-end or end-to-side techniques, employed between 1978 and 2004, exhibited lower patency rates of 61.1% [31]. The superiority of LIVE lies in its unique approach, involving suturing the epididymal tube longitudinally with two stitches and creating an adjustable opening for a secure and unobstructed anastomotic passage. Unlike other methods, LIVE allows flexibility in adjusting the diameter of the epididymal duct according to the vas deferens, ensuring enhanced security and unimpeded flow. Additionally, LIVE provides robust posterior wall support, further enhancing the procedure’s safety.

Our study adds valuable evidence to the existing literature supporting the efficacy of LIVE for treating EOA, which is further confirmed by the high rates of patency observed in our analysis in restoring sperm flow and enhancing fertility outcomes. With its technical complexity and superior results, LIVE has gained recognition as a highly esteemed procedure in the field of microsurgery for male infertility.

After confirming the efficacy of DA-LIVE and SA-LIVE in treating EOA, we conducted a comparative analysis to examine their differences. Our evaluation of postoperative patency rates demonstrated a mean patency rate of 65% for DA-LIVE and a slightly higher mean patency rate of 69% for SA-LIVE. However, it is important to highlight that there was no statistically significant difference in patency rates between the two techniques, regardless of the definition of patency employed. Our findings are similar to those of Wan et al. [12] in showing that the overall mean patency rate of patients in the SA-LIVE group was higher than that of patients in the DA-LIVE group (68% vs. 66.3%). The possible reasons for these differing findings may be attributed to variations in patient characteristics across the included studies and the growing number of recent publications specifically investigating SA-LIVE. Based on our findings, we can conclude that both techniques demonstrate similar effectiveness in achieving postoperative patency outcomes for patients with EOA. The SA-LIVE technique is suggested as a potential alternative surgical option when high−quality double−needle sutures are not easily accessible.

Hayden et al. [1], suggested that using a single-armed 10–0 suture during SA-LIVE may lead to lower patency rates than conventional DA-LIVE. Theoretically, DA-LIVE should have better postoperative patency rates because of its double-armed sutures, minimizing the risk of back-walling injury to the vas deferens. We agree with Hayden’s viewpoint, as SA-LIVE employs an "external-to-internal" needle insertion approach. Although adjusting the needle position can decrease the chance of unintentional suturing to the contralateral mucosa of the vas deferens, it cannot entirely eliminate this risk.

We evaluated the patency time postoperatively in LIVE procedures. Prolonged waiting times can lead to anxiety among patients and their families [19]. This may impact patients’ decisions regarding whether to opt for continue waiting or ART. In our analysis, the mean time for postoperative patency after LIVE procedures was approximately 4.63 months, irrespective of the specific criteria used to define patency. Specifically, DA-LIVE showed a patency time of 4.49 months, while SA-LIVE exhibited a slightly longer patency time of 4.84 months. However, no statistical difference was observed between these two techniques. Therefore, a common occurrence of delayed patency in SA-LIVE and DA-LIVE, indicating the need for a minimum waiting period of 4 months following LIVE for patients with EOA before considering alternative ART.

Furthermore, we evaluated the semen parameters following the successful of patency, as the quality of sperm has a significant impact on the partner’s pregnancy rate. Our analysis showed that the post-LIVE mean sperm concentration reached 26.90 million/ml. However, the mean sperm motility, which measured 23.74%, remained below the threshold of 32% defined by the fifth edition of the World Health Organization’s semen analysis standards. The findings of low sperm motility may indicate that the postoperatively shortened length of the epididymis may affect sperm maturation within the epididymis. Furthermore, the included literature provided no specific details regarding the timing of semen analysis after the procedure. Further investigations are required to better understand the potential impact of timing on semen analysis parameters.

After conducting our analysis, we found that the overall mean natural pregnancy rate for LIVE procedures was 38% during a follow-up period of 2–48 months. Specifically, the mean natural pregnancy rates for DA-LIVE and SA-LIVE were 34% and 36%. No significant differences were observed between the two techniques regarding natural pregnancy rates, suggesting their equal effectiveness in achieving natural pregnancy for individuals with EOA. However, it is crucial to consider factors beyond the specific surgical method utilized, such as the age of the patient’s partner, as they can also influence natural pregnancy rates [18].

There are several limitations to this analysis. First, the role of factors such as spouse’s age and spouse’s fertility assessment was not adequately accounted for in the included studies, which may have influenced the outcomes. Additionally, the follow−up time after MVE was variable among the included studies, which may have introduced variability in the reported outcomes. Another limitation is that unless performed by the same surgeon, the value of the information from these studies is limited to demonstrating similarity of SA-LIVE outcomes to DA-LIVE in very specific hands. This is merely for academic purposes. Furthermore, it’s essential to acknowledge that there were variations in the testing methods used across the different study centers. This inconsistency could potentially impact the results of our meta-analysis.

In conclusion, no statistical difference was observed between DA-LIVE and SA-LIVE regarding patency rate, patency time, semen quality, and natural pregnancy rate. These findings suggest that both techniques are effective and well−established for treating EOA. In situations where obtaining high-quality double-needle sutures proves challenging, the SA-LIVE technique emerges as a potential alternative surgical option. This approach offers a viable solution for cases where the availability or quality of such sutures may be limited.

## Supporting information

S1 File Descriptions of the parts of the excluded articles. Articles not included in the analysis consisted of those not based on 2-suture LIVE (n = 41), those with unavailable data (n = 2), and those involving animal studies (n = 3).
(DOCX)

S2 File The PRISMA 2020 statement. (DOCX)
