# Supplementary material for: Comparative evaluation of double- and single-armed two-suture longitudinal intussusception techniques in microsurgical vasoepididymostomy: An updated systematic review and meta-analysis
Source: PLoS One. 2024 Feb 5;19(2):e0298019. doi: 10.1371/journal.pone.0298019 (PMC10843031; doi:10.1371/journal.pone.0298019)
Supplement: S1 File — Articles not included in the analysis consisted of those not based on 2-suture LIVE (n = 41), those with unavailable data (n = 2), and those involving animal studies (n = 3). (DOCX) [file pone.0298019.s001.docx]

There were 70 records remaining after eligibility assessment. Out of these, 24 articles were included in the meta-analysis and detailed information can be found in the main text. The additional material mainly provides descriptions of the parts of the excluded articles. Articles not included in the analysis consisted of those not based on 2-suture LIVE (n=41), those with unavailable data (n=2), and those involving animal studies (n=3).

**Not based on 2-suture LIVE (n=41)**

1. Zhang Z, Zhang Y, Zhang N. Clinical outcome of microsurgical vasoepididymostomy versus epididymal or testicular sperm retrieval combined with intracytoplasmic sperm injection in obstructive azoospermia males. Andrologia. 2022 Sep;54(8):e14458.
2. Liu Z, Ding Z, Jiang H, Yuan Q, Xiao K, Wang B. A Modified Transversal Two-Suture Microsurgical Intussusception Vasoepididymostomy for the Treatment of Epididymal Obstructive Azoospermia. Eur Surg Res. 2023;64(2):246-251.
3. Shiraishi K, Matsuyama H. Outcomes of partial intussusception and endo-to-side vasoepididymostomy in men with epididymal obstructive azoospermia. Int J Urol. 2020 Dec;27(12):1124-1129.
4. Tiwari DP, Razik A, Das CJ, Kumar R. Prospective analysis of factors predicting feasibility & success of longitudinal intussusception vasoepididymostomy in men with idiopathic obstructive azoospermia. Indian J Med Res. 2019 Jan;149(1):51-56.
5. D. Pushkar, N. Akhvlediani, A. Bernikov and I. Reva. Follicle-stimulating hormone therapy after microsurgical vasoepididymostomy. Journal of Urology. 2019 Vol. 201(4):e770-e771.
6. G. Chiriacò, V. Modgil, H. M. Assiri, G. A. Blecher, H. Alnajjar, P. Sangster, et al. Epididymovasostomy: Patency, pregnancy rate and predictive factors for success in 109 patients over a decade. European Urology, Supplements 2019 Vol. 18(1):e331.
7. Y. Zhang, X. Yang, X. Wu, J. Feng, S. Chen and H. Zhang. Vasal vessels preserving microsurgical vasoepididymostomy. Journal of Sexual Medicine. 2017 Vol. 14(1):S110-S111.
8. C. S. Patro, N. C. Behera, P. C. Majhi, S. Swain, S. Panda, P. K. Mohanty, et al. Microsurgical vasoepididymostomy: A single center experience. Indian Journal of Urology. 2017 Vol. 33
9. C. H. Lu, W. J. S. Huang, I. S. Huang, A. T. L. Lin and K. K. Chen. The outcomes of scrotal exploration with vasoepididymostomy in patients with non-iatrogenic, non-traumatic obstructive azoospermia. Journal of Urology. 2017 Vol. 197(4):e1340-e1341
10. R. Flannigan, P. V. Bach, A. Ayangbesan, A. Gottesdiener and M. Goldstein. Timing of return of sperm to the ejaculate and late failures following vasal reconstruction. Journal of Urology. 2017 Vol. 197(4):e1209
11. Alom M, Ziegelmann M, Savage J, Miest T, Köhler TS, Trost L. Office-based andrology and male infertility procedures-a cost-effective alternative. Transl Androl Urol. 2017 Aug;6(4):761-772.
12. F. Neto, A. Ayangbesan, B. Najari, P. Bach, A. Gottesdiener, P. Li, et al. Comparing vasoepididymostomy technique outcomes: Longitudinal intussusception vasoepididymostomy (LIVE) versus other techniques. Journal of Urology. 2016 Vol. 195(4):e223
13. B. B. Najari, P. Bach, F. Neto, P. S. Li, M. Goldstein, A. J. Gottesdiener, et al. Longitudinal intussusception vasoepididymostomy learning curve. Fertility and Sterility. 2016 Vol. 106:e291-e292
14. B. Najari, A. Ayangbesan, A. Gottesdiener, P. Bach, F. T. L. Neto, P. Li, et al. Men undergoing vasoepididymostomy for vasectomy reversal have worse outcomes than men with primary epididymal obstruction. Journal of Urology. 2016 Vol. 195(4):e223-e224
15. H. Jiang, Q. Yuan, Z. Liu, J. Guo and Z. Zhou. A modified microscopic vasoepididymostomy to treat epididymal obstruction. Andrology. 2016 Vol. 4:111-112
16. Taniguchi H, Iwamoto T, Ichikawa T, Nagai A, Okada H, Fujisawa M, Tsujimura A, Shiraishi K, Hibi H, Nagao K, Iwasaki A, Kamba T, Tomomasa H, Takada S, Matsuda T; Male Infertility Surgical Forum in Japan. Contemporary outcomes of seminal tract re-anastomoses for obstructive azoospermia: a nationwide Japanese survey. Int J Urol. 2015 Feb;22(2):213-8.
17. B. K. Mahapatro, S. Choudhuri, J. J. Mishra, S. S. Pand, S. Swain, G. P. Singh, et al. Microsurgical vasoepididymostomy: A single center experience. Indian Journal of Urology. 2015 Vol. 31:S74-S75
18. Hussein A. A new one-layer epididymovasostomy technique. BJU Int. 2015 Apr;115(4):653-8.
19. A. Saxena and S. Garanayak. Robot assisted vasoepididymal anastomosis: Our initial experince of five cases. Indian Journal of Urology. 2014 Vol. 30:S98
20. P. Patnaik, S. Rathi, A. Agarwal, D. Shaw, S. Trivedi and U. S. Dwivedi. Microsurgical vasoepididymostomy-In Era of ICSI. Indian Journal of Urology 2014 Vol. 30:S100
21. Jiang HT, Yuan Q, Liu Y, Liu ZQ, Zhou ZY, Xiao KF, Yang JG. Multiple advanced surgical techniques to treat acquired seminal duct obstruction. Asian J Androl. 2014 Nov-Dec;16(6):912-6.
22. Harza M, Voinea S, Ismail G, Gagiu C, Baston C, Preda A, Manea I, Priporeanu T, Sinescu I. Predictive factors for natural pregnancy after microsurgical reconstruction in patients with primary epididymal obstructive azoospermia. Int J Endocrinol. 2014;2014:873527.
23. J. W. Goossen, A. A. Raheem, F. De Luca, A. N. Christopher and D. J. Ralph. Microsurgical epididymovasostomy outcomes for obstructive azoospermia. BJU International 2014 Vol. 113:1
24. S. Choudhuri, S. Swain, P. K. Mohanty, S. S. Dash, J. J. Mishra, G. P. Singh, et al. Microsurgical vasoepididymostomy : A single center experience. Indian Journal of Urology 2014 Vol. 30:S99
25. Y. Zhang, H. Zhang, W. Huang, X. Liu and X. Gao. Microsurgical 2-suture intussusception vasoepididymostomy: Transversal or longitudinal?. Journal of Sexual Medicine 2013 Vol. 10:225
26. Y. Zhang, H. Zhang, C. Hu, W. T. Huang, X. Liu, L. Y. Li, et al. Modified intussusception vasoepididymostomy: Does it has benefits?. Journal of Urology 2013 Vol. 189(4):e654
27. Zhang H, Huang WT, Ruan XX, Li LY, DI JM, Liu XP, Xiao HJ, Gao X, Zhang Y. Microsurgical transverse 2-suture intussusception vasoepididymostomy: effectiveness and rationality. Chin Med J (Engl). 2013;126(24):4670-3.
28. Safarinejad MR, Lashkari MH, Asgari SA, Farshi A, Babaei AR. Comparison of macroscopic one-layer over number 1 nylon suture vasovasostomy with the standard two-layer microsurgical procedure. Hum Fertil (Camb). 2013 Sep;16(3):194-9.
29. P. Ping, X. Chen, Y. Dong, K. Sun, Y. Liu, L. Zhou, et al. Use of single-armed suture microsurgical vasoepididymostomy in the treatment of obstructive azoospermia. Chinese Journal of Andrology. 2012 Vol. 26(5):36-39
30. X. D. Li, Q. X. He, S. H. Fan, Z. Y. Jiang and Z. X. Wang. [Diagnosis and treatment of epididymal obstructive azoospermia by microsurgery]. Zhonghua nan ke xue = National journal of andrology 2012 Vol. 18(7):611-614
31. A. M. Abdel Raheem, G. Garaffa, M. S. Shabir, E. Zacharakis, A. M. Muneer and S. Minhas. The outcome of microsurgical epididymovasostomy in the management of obstructive azoospermia. BJU International 2012 Vol. 109:12
32. Smrkolj T, Virant-Klun I, Sinkovec J, Oblak C, Zorn B. Epididymovasostomy as the first-line treatment of obstructive azoospermia in young couples with normal spermatogenesis. Reprod Biomed Online. 2010 May;20(5):594-601.
33. X. Tu, L. Zhao, L. Deng, W. Wang, L. Zhao, H. Liang, et al. The diagnosis and surgical treatment for obstructive azoospermia: Report of 56 cases. Urology 2009 Vol. 74(4):S251-S252
34. G. Zhang, W. Bai, K. Xu, Y. Wang, X. Wang, S. Deng, et al. Intussusception vasoepididymostomy for the treatment of obstructive azoospermia. Chinese Journal of Andrology 2008 Vol. 22(4):50-52
35. J. Pryor. Prospective analysis of outcomes after microsugical intussusception vasoepididymostomy. European Urology 2006 Vol. 49(1):197-198
36. Ho KL, Witte MN, Bird ET, Hakim S. Fibrin glue assisted 3-suture vasovasostomy. J Urol. 2005 Oct;174(4 Pt 1):1360-3; discussion 1363.
37. Chan PT, Brandell RA, Goldstein M. Prospective analysis of outcomes after microsurgical intussusception vasoepididymostomy. BJU Int. 2005 Sep;96(4):598-601.
38. P. V. Bach, B. B. Najari, F. Neto, A. Ayangbesan, A. V. Gottesdiener and M. Goldstein. Repeat microsurgical reconstruction after failed initial vasectomy reversal. Fertility and Sterility 2016 Vol. 106:e293
39. K. Ostrowski, N. Tadros, J. Hedges and E. Fuchs. The significance of light microscopy findings at the time of vasoepididymostomy. Journal of Urology 2015 Vol. 193(4):e939-e940
40. A. Gudeloglu, J. Brahmbhatt, T. Patel, K. Priola and S. Parekattil. Robotic assisted re-do vasectomy reversal for previously failed pure microsurgical reversal. European Urology, Supplements 2013 Vol. 12(1):e841
41. S. J. Parekattil, A. Gudeoglu, J. Brahmbhatt, K. B. Priola and M. S. Cohen. Robotic assisted versus pure microsurgical vasectomy reversal: Prospective control trial. Fertility and Sterility 2011 Vol. 96(3):S230-S231

**Without available data (n=2)**

1. N. S. Voinea, C. Gagiu, S. Nedelea, I. Manea, A. Preda, C. Gingu, et al. A prospective comparative study of two reconstruction techniques in patients with obstructive azoospermia. European Urology, Supplements 2014 Vol. 13(6):e1234
2. Schwarzer JU. Vasectomy reversal using a microsurgical three-layer technique: one surgeon's experience over 18 years with 1300 patients. Int J Androl. 2012 Oct;35(5):706-13.

**Animal studies (n=3)**

1. Y. Yuan, H. Lei, Z. Zhang, J. Peng, W. Cui and Z. Xin. Guo's single-armed suture technique for microsurgical vasoepididymostomy. Andrology. 2018 Vol. 6 :77-78.
2. Y. Yuan, Y. Tang, Z. Zhang, Z. Xin and Y. Guo. Guo's single-armed suture technique for microsurgical vasoepididymostomy. Journal of Sexual Medicine. 2017 Vol. 14(1):S120
3. Monoski MA, Schiff J, Li PS, Chan PT, Goldstein M. Innovative single-armed suture technique for microsurgical vasoepididymostomy. Urology. 2007 Apr;69(4):800-4.
